# Reconsidering the generation time hypothesis based on nuclear ribosomal *ITS *sequence comparisons in annual and perennial angiosperms

**DOI:** 10.1186/1471-2148-8-344

**Published:** 2008-12-29

**Authors:** David F Soria-Hernanz, Omar Fiz-Palacios, John M Braverman, Matthew B Hamilton

**Affiliations:** 1 Department of Biology, Georgetown University, Washington DC, USA; 2 Real Jardin Botanico de Madrid, CSIC, Madrid, Spain; 3 The Genographic Project, National Geographic Society, Washington DC, USA; 4 Department of Life Sciences, Imperial College London, Ascot, UK

## Abstract

**Background:**

Differences in plant annual/perennial habit are hypothesized to cause a generation time effect on divergence rates. Previous studies that compared rates of divergence for internal transcribed spacer (*ITS*1 and *ITS*2) sequences of nuclear ribosomal DNA (nrDNA) in angiosperms have reached contradictory conclusions about whether differences in generation times (or other life history features) are associated with divergence rate heterogeneity. We compared annual/perennial *ITS *divergence rates using published sequence data, employing sampling criteria to control for possible artifacts that might obscure any actual rate variation caused by annual/perennial differences.

**Results:**

Relative rate tests employing *ITS *sequences from 16 phylogenetically-independent annual/perennial species pairs rejected rate homogeneity in only a few comparisons, with annuals more frequently exhibiting faster substitution rates. Treating branch length differences categorically (annual faster or perennial faster regardless of magnitude) with a sign test often indicated an excess of annuals with faster substitution rates. Annuals showed an approximately 1.6-fold rate acceleration in nucleotide substitution models for *ITS*. Relative rates of three nuclear loci and two chloroplast regions for the annual *Arabidopsis thaliana *compared with two closely related *Arabidopsis *perennials indicated that divergence was faster for the annual. In contrast, *A. thaliana ITS *divergence rates were sometimes faster and sometimes slower than the perennial. In simulations, divergence rate differences of at least 3.5-fold were required to reject rate constancy in > 80 % of replicates using a nucleotide substitution model observed for the combination of *ITS*1 and *ITS*2. Simulations also showed that categorical treatment of branch length differences detected rate heterogeneity > 80% of the time with a 1.5-fold or greater rate difference.

**Conclusion:**

Although rate homogeneity was not rejected in many comparisons, in cases of significant rate heterogeneity annuals frequently exhibited faster substitution rates. Our results suggest that annual taxa may exhibit a less than 2-fold rate acceleration at *ITS*. Since the rate difference is small and *ITS *lacks statistical power to reject rate homogeneity, further studies with greater power will be required to adequately test the hypothesis that annual and perennial plants have heterogeneous substitution rates. *Arabidopsis *sequence data suggest that relative rate tests based on multiple loci may be able to distinguish a weak acceleration in annual plants. The failure to detect rate heterogeneity with *ITS *in past studies may be largely a product of low statistical power.

## Background

Comparative studies of molecular substitution rates between lineages provide insights into the mechanisms that cause evolution of DNA sequences. Under the neutral theory [1,2] rates of nucleotide substitutions are expected to be equal to rates of mutation, thus a constant rate of nucleotide substitution in homologous DNA sequences should be observed among lineages that share mutation rates. Neutral theory assumes that genetic drift is the primary evolutionary mechanism causing molecular evolution and predicts that rates of sequence change would be both constant over time and independent of the effective population size. Heterogeneity in substitution rates can be explained under neutral theory by either unevenness of mutation rates at individual loci (manifested as locus effects) or correlated mutation rates across all loci within species (manifested as lineage effects). Alternatively, natural selection may cause rate heterogeneity among loci and lineages via purifying selection that reduces the probability of substitution due to functional constraint or through the increased probability of substitution associated with positive natural selection [3-6]. Identifying causes of rate heterogeneity as well as specific variables that affect underlying mutation and substitution rates is fundamental to understanding the mechanisms that cause evolution of DNA sequences (reviewed in [7]).

Differences in generation time could affect substitution rates, causing lineage effects on substitution rates if organisms with shorter generation times experience more mutations per unit of chronological time than organisms with longer generation times. This neutral explanation for rate heterogeneity among lineages is commonly called the generation time hypothesis. Under the generation time hypothesis, lineage-specific heterogeneity in rates of divergence can be explained by differences in the number of germ line cell divisions per unit time among lineages that otherwise share constant mutation rates. Therefore, under the generation time hypothesis substitution rates are expected to be negatively correlated with generation time [5,8,9]. Generation time effects on synonymous substitution rates have been widely observed at multiple loci for several mammalian species [2,3,9-15]. Generation-time-like effects have also been tested for in organism such as RNA viruses where faster substitution rates were correlated with higher frequencies of replication [16] and in spore-forming bacteria where rates of divergence were not related to spore dormancy [17].

In angiosperms, expected generation time impacts on rates of molecular evolution are not as clear as in animals since plants lack distinct germ and somatic cell lines. Plant cells are totipotent and the number of cell divisions between germination and gamete production can vary from individual to individual and even among parts of a single individual. The generation time hypothesis modified for plants assumes that variation in the frequency of cell replication is correlated with differences in annual/perennial habit. Since annuals have shorter minimum time to first flowering than perennials, it has been assumed that annuals would also experience a higher frequency of cell replication per chronological time and thereby a faster rate of divergence when compared to perennials [18]. The generation time hypothesis has been invoked to explain why annual species exhibited higher rates of molecular evolution than perennial species for several nuclear, mitochondrial and chloroplast loci (e.g. [19-23]). However, results from studies that support a generation time effect in plants have two primary limitations [24]. First, some studies used multiple non-independent comparisons in their analyses that may lead to statistical difficulties as well as potential phylogenetic bias. Second, the taxa compared were highly divergent so that other evolved differences in addition to generation time could also have caused the rate variation observed. Comparing divergence rates in phylogenetically-independent sets of annual/perennial pairs that are recently diverged can correct for these two pitfalls when testing for a generation time effect in angiosperms [24].

Loci that can be used to estimate divergence rates are limited in the vast majority of angiosperms, which restricts comparisons of substitution rates in multiple independent sets of recently diverged plant taxa. For example, the plant mitochondrial genome exhibits a fast pace of structural evolution but the lowest rate of nucleotide substitutions of all three plant genomes making it especially difficult to obtain sequences in multiple plant lineages with sufficient divergence [18,25-28]. Universal primers are available for multiple chloroplast regions but, like mitochondrial regions, the utility of these regions is often limited by low sequence divergence at shallow phylogenetic relatedness. Nuclear loci are not widely available in multiple plant lineages since nuclear genomes have variable architecture, abundant multigene families with rapid duplication and loss complicating the identification of orthologous loci [18,27]. There is also a wide range of substitution rates among nuclear DNA sequences in plants [29], requiring multiple loci in comparative studies to average rates over independent loci.

The internal transcribed spacers (*ITS*1 and *ITS*2) of nuclear ribosomal DNA (nrDNA) are the only nuclear DNA markers currently available for comparative tests of the generation time hypothesis in a broad range of recently diverged plant taxa for several reasons. First, *ITS *regions are universally amplifiable in plants and many plant taxa have been sequenced. Second, *ITS *regions are highly variable at the nucleotide level. Third, it is commonly believed that *ITS *multicopy arrays are homogenized by concerted evolution so that intraspecific polymorphism does not complicate estimates of divergence [30,31]. Moreover, *ITS *regions have been used extensively in molecular evolution studies of plants such as demonstrating that rates of *ITS *nucleotide substitution are associated with species diversity [32], reproductive isolation and life history [33], and environmental variables ([33]; but see [34]).

Two recent studies compared rates of *ITS*1 and *ITS*2 divergence using phylogenetically independent sets of angiosperms differing in life history but reached opposite conclusions about whether differences in life history affect rates of divergence. In the first study, Whittle and Johnston [24] did not find an association between relative rates of nucleotide substitution and annual/perennial life history in 22 species pairs, leading them to conclude that the generation time hypothesis does not apply to angiosperms. In another recent study, clades with a predominantly herbaceous life history exhibited an almost twice-faster average rate of divergence than predominantly long-lived woody clades using 28 independently calibrated absolute rates of *ITS *nucleotide substitution [35]. Both studies consistently did not reject the null hypothesis of constant divergence rates when comparing life histories. Since low statistical power of rate tests was suspected, both papers also treated substitution rate differences qualitatively or categorically (e.g. annual is faster or perennial is faster regardless of the magnitude of the rate difference). These conflicting results mandate further research into whether differences in generation times are correlated with substitution rates in angiosperms.

Given that *ITS*1 and *ITS*2 are currently among the only sequences available to test for rate heterogeneity among a wide sampling of plant taxa, it is essential to assess the statistical power of rate heterogeneity tests based on *ITS *sequences. It is critical to determine the magnitude of rate heterogeneity required to reliably reject the null hypothesis of rate constancy when evaluating whether differences in annual/perennial habit have heterogeneous substitution rates. Low statistical power will result in type II errors (incorrectly failing to reject the null hypothesis of rate constancy) that could lead to an erroneous conclusion that annual/perennial habit is not associated with divergence rates. One main cause of low statistical power is a small number of nucleotide substitutions available to estimate divergence, a common situation when recently diverged species are being compared. Simulations have shown that the power of Tajima's relative rate test [36], distance-based relative rate tests [9], and the maximum-likelihood relative ratio test [6] are all dependent on sequence lengths, the relatedness of the outgroup taxa, and the employment of an appropriate model of nucleotide substitution [37,38]. The alternative approach of categorical treatment of substitution rate differences in annual/perennial comparisons is based on the assumption that the direction of rate differences would accurately test rate heterogeneity. However, this approach has not yet been subjected to a rigorous power analysis.

In this article, we test whether annual/perennial habit affects rates of divergence by comparing both relative rates of molecular evolution and categorical branch length differences in 16 independent annual/perennial species pairs. *ITS*1 and *ITS*2 sequences were obtained from GenBank under strict sampling criteria designed to control for artifacts contributing additional variation in divergence rates that could obscure any rate variation caused by differences in life history. The criteria were that each annual/perennial pair was recently diverged, had at least eight nucleotide changes between taxa, had *ITS *sequences for two outgroup taxa available, and the *ITS *sequences were originally obtained from a single PCR amplicon. The power of maximum likelihood relative rate tests was investigated by determining the degree of rate heterogeneity required to reliably reject rate constancy for DNA sequences simulated under average nucleotide substitution parameters of *ITS *sequences. We also used simulations to assess whether categorical treatment of branch length differences is an appropriate method to test for rate heterogeneity when a relative rate test does not reject rate constancy. In addition, we utilized sequences of three nuclear loci, two chloroplast regions and multiple intra-specific nrDNA ribotypes for the annual *Arabidopsis thaliana *and two closely related perennials (*Arabidopsis lyrata *subsp. *lyrata *and *A. lyrata *subsp. *petrea*) to test whether substitution rate differences at the *ITS *regions were correlated across multiple loci as expected under the generation time hypothesis.

## Results

### *ITS *annual/perennial substitution rates

The edited sequences had between 210–267 sites for *ITS*1, between 183–257 sites for *ITS*2 and between 420–505 sites for the combined *ITS *region. Results from the maximum-likelihood relative rate test and the categorical treatment of branch length differences, as well as the estimated rate differences in 16 independent annual/perennial comparisons for the *ITS*1, *ITS*2 and combined *ITS *data using two outgroup taxa, are summarized in Table 1. The maximum-likelihood relative rate test rejected the null hypothesis of rate constancy in 12 out of 48 comparisons with less divergent outgroups and in 13 out of 48 comparisons with more divergent outgroups. In most tests where the null was rejected, annual species exhibited faster rates of nucleotide substitution than perennial species (10 annuals versus 2 perennials with less divergent outgroups and 8 annuals versus 5 perennials with more divergent outgroups). Similar results for relative rate tests were obtained with Tajima's 1D relative rate test (results not shown). After treating branch length differences categorically, a sign test showed a significant excess of faster substitution rates in annual taxa for all *ITS *sequence regions with both more and less diverged outgroups with the exception of *ITS*1 using more diverged outgroups (Table 1). In summary, the results indicated rate homogeneity for many comparisons but annuals more frequently exhibited significantly faster rates of nucleotide substitution when there was significant rate heterogeneity.

**Table 1:** Relative branch lengths for *ITS*1, *ITS*2 and combined *ITS *sequences between comparisons of recently diverged annual (before the slash) and perennial (after the slash) species when two different phylogenetically related outgroup taxa are used.

Taxa^a^	*ITS*1	Rate Δ^b^	A/P^c^	*ITS*2	Rate Δ	A/P	*ITS*	Rate Δ	A/P
*Arabidopsis*	0.042/0.033	1.30	A	0.034/0.034	1	=	0.039/0.033	1.17	A
	
	0.047/0.029	1.59	A	0.041/0.021	1.90	A	0.044/0.026	1.66	A

*Astragalus*	0.038/0.033	1.15	A	0.045/0.033	1.37	A	0.043/0.034	1.28	A
	
	0.039/0.034	1.15	A	0.043/0.038	1.15	A	0.041/0.036	1.14	A

*Lupinus*	**0.044**/0.009	4.91	A	0.021/0.015	1.35	A	0.032/0.013	2.53	A
	
	0.033/0.021	1.55	A	0.021/0.016	1.33	A	0.028/0.017	1.63	A

*Bellis*	0.012/0.004	3.35	A	0.015/0.000	-	A	**0.014**/0.001	9.20	A
	
	0.012/0.004	3.30	A	0.010/0.005	2.11	A	0.011/0.004	2.70	A

*Erigeron*	0.018/0.010	1.86	A	0.045/0.012	3.64	A	0.029/0.011	2.65	A
	
	0.011/0.019	1.74	P	0.005/**0.058**	11.7	P	0.006/**0.039**	6.53	P

*Machaeranthera*	0.028/0.015	1.92	A	0.024/0.009	2.56	A	0.026/0.012	2.17	A
	
	0.044/0.013	3.36	A	0.004/0.031	7.76	P	0.016/0.023	1.45	P

*Claytonia*	0.080/0.024	3.30	A	**0.065**/0.006	10.0	A	**0.075**/0.014	5.30	A
	
	**0.083**/0.019	4.43	A	**0.063**/0.013	5.01	A	**0.073**/0.015	4.78	A

*Collomia*	0.017/0.029	1.79	P	0.014/0.019	1.35	P	0.016/0.024	1.44	P
	
	0.008/**0.039**	4.98	P	0.019/0.014	1.35	A	0.014/0.027	1.90	P

*Erodium*	0.032/0.007	4.72	A	0.021/0.015	1.40	A	0.025/0.012	2.11	A
	
	0.031/0.009	3.48	A	0.026/0.011	2.39	A	0.029/0.009	3.16	A

*Linanthus*	**0.068/**0.012	5.81	A	0.049/0.043	1.15	A	0.057/0.027	2.13	A
	
	**0.071**/0.008	8.55	A	0.039/0.054	1.38	P	0.055/0.029	1.85	A

*Nicotiana*	0.033/0.017	1.97	A	0.035/0.029	1.20	A	0.033/0.023	1.47	A
	
	0.039/0.010	3.96	A	0.023/0.039	1.71	A	0.032/0.024	1.33	A

*Potentilla*	0.037/0.059	1.59	P	0.026/0.021	1.24	A	0.033/0.041	1.26	P
	
	0.038/0.054	1.42	P	0.037/0.008	4.79	A	0.038/0.033	1.15	A

*Ranunculus*	0.019/0.022	1.08	P	0.025/0.019	1.27	A	0.023/0.021	1.08	A
	
	0.009/0.029	3.15	P	0.039/0.006	6.93	A	0.023/0.019	1.21	A

*Sanicula*	0.01/0.031	3.20	P	0.013/**0.119**	9.27	P	0.011/**0.074**	6.67	P
	
	0.007/0.031	4.17	P	0.022/**0.114**	5.12	P	0.015/**0.071**	4.70	P

*Sidalcea*	0.034/0.019	1.79	A	**0.077**/0	N/A	A	**0.056**/0.008	6.68	A
	
	0.030/0.019	1.56	A	**0.063**/0.005	11.9	A	**0.045**/0.013	3.57	A

*Vulpia/Festuca*	**0.060**/0.009	6.35	A	**0.075**/0.009	8.70	A	**0.068**/0.010	7.02	A
	
	0.058/0.012	4.89	A	**0.067**/0.009	7.30	A	**0.061**/0.012	5.09	A
	
Number of annuals exhibiting			12 (0.038)			13 (0.011)			12 (0.038)
	
longer branch lengths^d^			11 (0.105)			12 (0.038)			11 (0.105)

^a ^Taxa used for each comparison (see Table 4).

^b ^Rate difference (Rate Δ) between ingroup taxa was calculated by dividing the taxon exhibiting the faster substitution rate (longer branch length) by the taxon with the slower substitution rate (shorter branch length).

^c ^A indicates the annual had a faster substitution rate than perennial, P indicates the perennial had a higher substitution rate and = indicates equal rates.

^d ^*p*-values of a one-tailed sign test with a null hypothesis of equal frequency are indicated in parentheses

The first row of each annual/perennial comparison gives the estimated substitution rate (or branch length) with the less divergent outgroup and the second row gives the estimates for the more divergent outgroup.

Bold entries indicate statistically significant rate heterogeneity by the maximum likelihood relative rate test.

Using two outgroup taxa with different levels of divergence in each annual/perennial comparison showed that substitution rates varied slightly but did not change the general conclusion that annuals exhibited faster rates of substitution than perennials. For *ITS*1 using less divergent outgroups, relative rate tests rejected rate constancy in three of 16 comparisons, indicating that three annual species exhibited a significantly faster rate of substitution. In the same way, annual taxa exhibited longer branch lengths in 12 of the 16 categorical comparisons (sign test, *p *= 0.038). When the same annual/perennial species pairs where compared using more divergent outgroups, two annuals and one perennial showed significantly faster rates by relative rate tests while 11 of 16 qualitative comparisons (sign test, *p *= 0.105) exhibited longer branch lengths for annual taxa. For the *ITS*2 data, four cases (three annuals and one perennial) rejected rate constancy when less divergent outgroups were employed and in 13 of 16 qualitative comparisons (sign test, *p *= 0.011) annuals showed longer branch lengths. If more divergent outgroups were used, five cases (three annuals and two perennials) rejected rate constancy and 12 of 16 qualitative comparisons (sign test, *p *= 0.038) exhibited longer branch lengths for annual taxa. For the combined *ITS *sequence data with less diverged outgroups, four annuals and one perennial species exhibited significantly faster substitution rates by relative rate tests and 13 of 16 qualitative comparisons (sign test, *p *= 0.011) exhibited longer branch lengths for the annual taxa. If more divergent outgroups were used with combined *ITS *sequence data, three annuals and two perennials rejected rate constancy and 12 of 16 qualitative comparisons (sign test, *p *= 0.038) exhibited longer branch lengths for annual taxa.

### Power simulations

**Figure 1:**
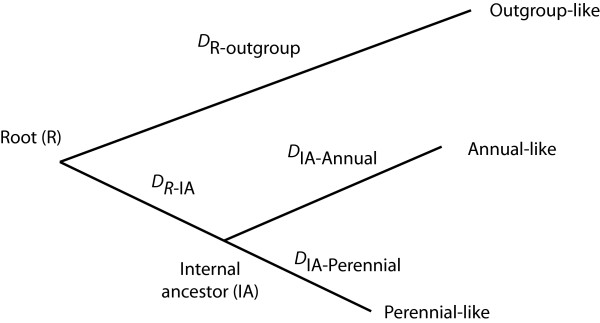
**Schematic of the tree topology used when simulating DNA sequence triplets for power analyses.** The program used to simulate DNA sequences (Seq-Gen) continues to add nucleotide changes until threshold divergence values have been reached. These threshold divergence values were obtained by averaging the estimated sets of branch lengths from actual *ITS* sequences for 16 annual/perennial/outgroup comparisons (Table 1). These averaged values are given in Phylip format in Table 2. The threshold divergence values for the perennial-like taxon (*D*_IA-perennial_) and the outgroup-like taxon (*D*_R-outgroup_, *D*_R-IA_) were kept constant for each outgroup (closer and further) and each set of nucleotide substitution parameters (*ITS*1-like, *ITS*2-like and Combined-*ITS*-like). To model rate heterogeneity, the threshold divergence value of the annual-like taxon (*D*_IA-annual_) for each set of replicate simulations was determined by multiplying the perennial-like taxon divergence threshold (*D*_IA-perennial_) by1.5 to 5 in steps of 0.5.

**Figure 2:**
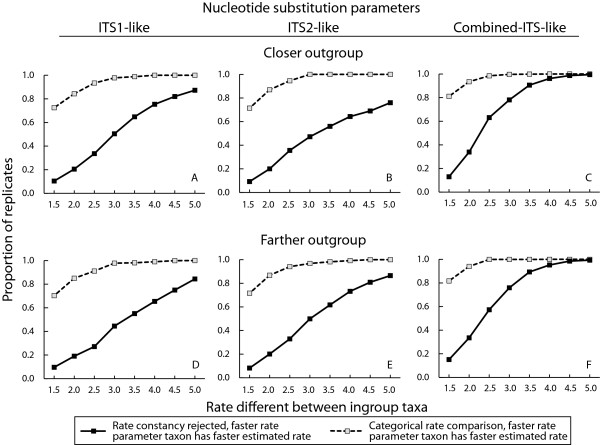
**The frequency of significant rate heterogeneity by maximum likelihood relative rate tests along with the frequency of rate heterogeneity indicated by categorical rate comparisons in simulated data.** Each data point represents the proportion of 1000 replicate simulated ITS-like sequence triplets with one of the ingroup taxon evolving with a substitution rate parameter between 1.5 and 5 times faster than the other ingroup taxon. In addition, the graphs show the proportion of 1000 replicates which correctly indicated that the taxon with the faster substitution rate parameter exhibited a higher substitution rate using qualitative substitution rate comparisons regardless of whether the maximum likelihood relative rate test rejected rate constancy. Simulated sequences were obtained with mean divergence and nucleotide substitution parameter sets estimated from actual ITS sequences (see Table 2).

The phylogeny used in the simulations is shown in Figure 1 and the nucleotide substitution parameter sets implemented in simulations are given in Table 2. The Kimura 2 parameter nucleotide substitution model (K80 or K2P), which assumes equal base frequencies and variable transition and transversion frequencies [39], was the one most frequently estimated for each of the 16 annual/perennial species pair comparisons for all six *ITS *sequence datasets (results not shown, substitution models available from the authors). Overall, the simulations indicated that a maximum likelihood relative rate test had an increasing chance to detect rate heterogeneity for *ITS*-like sequences as the rate difference between ingroup taxa increased (Figure 2). Relative rate tests rejected the null hypothesis in no more than 80% of replicate simulations for a given set or parameters for both *ITS*1-like and *ITS*2-like sequences, independently of the level of divergence of the outgroup used (see solid lines in Figure 2A, B, D and 2E). With a 3-fold or less rate difference between ingroup taxa, *ITS*1-like and *ITS*2-like simulated sequences rejected rate constancy for no more than 50% of replicates. For combined *ITS*-like sequences, the power of the test increased and rate constancy was rejected in about 80% of replicates with a 3.5-fold or greater rate difference between ingroup taxa (see solid lines in Figure 2C and 2F).

**Table 2:** Nucleotide substitution parameters estimated from *ITS *sequence data of 16 annual/perennial pairs used to simulate DNA sequences with Seq-Gen.

	*ITS*1	*ITS*2	Combined *ITS*
Phylip tree	((A:0.036, P:0.021): 0.006, O:0.113)	((A:0.036, P:0.024): 0.003, O:0.095)	((A:0.036, P:0.022): 0.004, O:0.103)
	((A:0.035, P:0.022): 0.012, O:0.186)	((A:0.033, P:0.028): 0.008, O:0.192)	((A:0.033, P:0.025): 0.010, O:0.185)

Ti/Tv	1.59	2.34	1.46
	2.53	2.25	1.48

Seq. length	237	214	450
	234	213	447

Rate Δ	1.73	1.52	1.63
	1.60	1.18	1.34

Phylip tree corresponds to the branch length thresholds and topology where A is the annual-like taxon, P is the perennial-like taxon and O is the outgroup.

Ti/Tv indicates the transition/transversion ratio, seq. length indicates the average sequence length and rate difference rate (Rate Δ is the average estimated substitution rate difference for each annual and perennial pair.

The top set of parameters in each row represents the less diverged outgroup and the bottom set in each row the more diverged outgroup.

The proportion of replicates where the faster evolving taxon had a qualitatively higher substitution rate was at least 70% even with a rate difference as low as 1.5-fold. Categorical rate comparisons identified the annual-like taxon as faster in 100% of replicates when the rate difference was 3-fold or greater for all three *ITS*-like sequences. The proportion of replicates with significant rate heterogeneity for each of the *ITS*-like sequences was similar between the more and less divergent outgroups.

**Figure 3:**
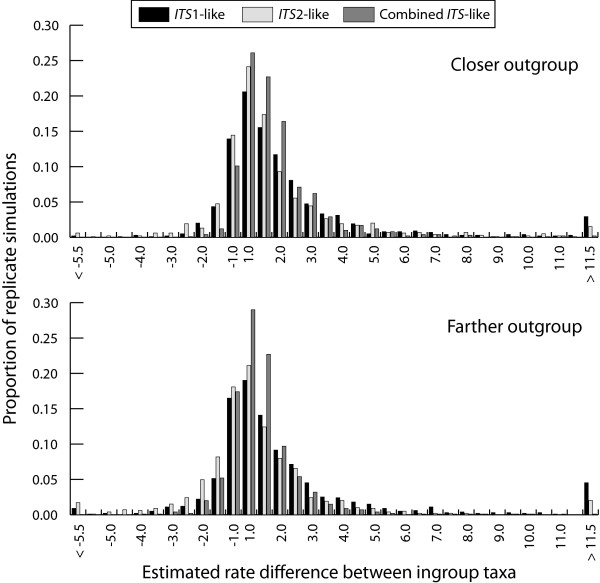
**Histograms of estimated substitution rate differences between annual-like and perennial-like species pairs in 1000 independent replicates that were simulated under the average nucleotide substitution parameter sets estimated from actual *ITS *sequences (see Table **2**).** The value axis gives the ratio of the faster substitution rate over the slower substitution rate. The rate difference ratio was assigned a negative value when the perennial-like taxon had a faster estimated substitution rate and was positive when the annual-like taxon had a faster estimated substitution rate.

The distributions of estimated rate differences between ingroup taxa among replicate sequence simulations for all six different nucleotide substitution model parameter sets (see Table 2) are shown in Figure 3. Estimated rate differences within a nucleotide substitution parameter exhibited high variation and extremely long tails for all six nucleotide substitution parameter sets. The mean and coefficient of variation were not appropriate to summarize the distributions because of non-normality (20 or so very extreme values at each tail lead to a large difference between the mean and mode). Medians and 95% confidence intervals (CI) of estimated substitution rate differences for replicate simulations were: median = 1.74, CI = -2.2 – 14.6 for *ITS*1-like sequences; median = 1.52, CI = -2.9 – 10.1 for *ITS*2-like sequences; median = 1.77, CI = -1.4 – 5.8 for combined-*ITS*-like sequences with a less diverged outgroup. Using a more diverged outgroup, replicate simulations exhibited rate difference distributions of median = 1.56, CI = -3.2 – 25.1 for *ITS*1-like sequences; median = 1.17, CI = -4.9 – 8.4 for *ITS*2-like sequences; median = 1.42, CI = -2.1 – 4.2 for combined-*ITS*-like sequences. The median value of the estimated rate differences was nearly identical in each case to the rate difference parameter in Table 2 and 95% CIs of estimated rate differences overlapped extensively.

### Arabidopsis annual/perennial substitution rates

Results of relative rate tests, estimates of substitution rates and estimated substitution rate differences between the annual *A. thaliana *and the perennials *A. lyrata *and *A. petraea *for various nrDNA ribotypes, the three nuclear loci and two chloroplast regions are summarized in Table 3. Rate constancy was rejected in a single instance, where the chloroplast region had a significantly faster substitution rate for the annual *A. thaliana*. Qualitatively, substitution rate differences estimated at each of the three nuclear loci were similar and uniformly indicated a faster rate for the annual species. The average substitution rate difference for all nuclear loci was 1.35-fold between *A. thaliana *and *A. lyrata *and 1.32-fold between *A. thaliana *and *A. petraea*.

**Table 3:** Estimated branch lengths and substitution rate differences (Rate Δ) for comparisons between the annual *Arabidopsis thaliana *and the two perennials *Arabidopsis lyrata *subspecies *lyrata *and *Arabidopsis lyrata *subspecies *petraea *using five nuclear loci (*ITS*1, *ITS*2, *Chs*, *Adh*, *Pgi*C), two chloroplast regions (*rbc*L and *matK*) and *Crucihimalaya himalaica *as the outgroup.

	*ITS*1	*ITS*2	*ITS*	*rbc*L	*matK*	*Chs*	*Adh*	*Pgi*C
*A. thaliana*	0.0399	0.0316	0.0382	0.0047	0.0121	0.0278	0.048	0.064

*A. lyrata*	0.0359	0.0316	0.0329	0.0015	0.0078	0.0227	0.033	0.046

Rate Δ	+1.11	1	+1.16	+3.13	+1.55	+1.22	+1.45	+1.39

*A. thaliana*	0.0392	0.0355	0.0395	0.0047	0.0113	0.0303	0.049	0.062

*A. petraea*	0.0527	0.0284	0.0418	0.0007*	0.0079	0.0253	0.035	0.046

Rate Δ	-1.34	+1.25	-1.06	+6.7	+1.43	+1.2	+1.4	+1.35

*A. thaliana*	0.0399	0.0355	0.0403					

*A. petraea*^a^	0.0354	0.0284	0.0311					

Rate Δ	+1.13	+1.25	+1.3					

* p < 0.05

^a ^This *ITS *ribotype for *A. petraea *corresponds to R2 in Table 5.

When the three nuclear loci were concatenated into a single sequence, a Tamura-Nei nucleotide substitution model with a gamma parameter was obtained (results not shown). When the entire concatenated nuclear sequence was used in the likelihood relative rate test, *A. thaliana *showed significantly faster divergence rates when compared to both *A. lyrata *(*p *= 0.017) and *A. petraea *(*p *= 0.025; results not shown). For the concatenated nuclear sequences, the average substitution rate difference was 1.35-fold between *A. thaliana *and *A. lyrata *and 1.33-fold between *A. thaliana *and *A. petraea*.

When the two chloroplast regions were concatenated into a single sequence, a Hasegawa-Kishino-Yano nucleotide substitution model best fit the data (results not shown). In contrast with the results from the concatenated nuclear sequences, relative rate tests did not reject the null hypothesis of rate constancy for the concatenated chloroplast sequences between *A. thaliana *and both *A. lyrata *(*p *= 0.093) and *A. petraea *(*p *= 0.092; results not shown). Categorical analyses for the concatenated chloroplast sequences indicated a faster divergence rate for *A. thaliana *when compared to *A. lyrata *(1.7-fold difference) and *A. petraea *(1.8-fold difference).

No relative rate tests rejected rate constancy for any comparison of *Arabidopsis ITS *sequences. For all *ITS *sequences in *Arabidopsis*, categorical treatment of substitution rates as well as estimated rate differences between annual and perennial taxa showed a roughly equal number of cases where the annual and the perennial exhibited a faster substitution rate. Sometimes the taxon with the faster rate for *ITS*1 had the slower rate for *ITS*2, such as when *A. petraea *(R1 ribotype) had a qualitatively higher substitution rate at *ITS*1 but qualitatively lower substitution rate at *ITS*2. In another case, using the alternative ribotype R2 for *A. petraea *both *ITS*1 and *ITS*2 exhibited qualitatively higher substitution rates in the annual taxon. When additional outgroups and nrDNA ribotypes were used in the annual/perennial/outgroup comparisons for *ITS *(data not shown), both *A. thaliana *and the perennial taxa had qualitatively faster substitution rates with about equal frequency. The alternating pattern of either the annual or perennial taxon exhibiting a faster estimated substitution rate for *ITS *sequences was in contrast to the consistent pattern of faster estimated substitution rates for the annual *A. thaliana *at the three nuclear loci and the two chloroplast regions.

## Discussion

Overall, the null hypothesis of rate constancy for *ITS *sequences was not rejected in the majority of annual/perennial comparisons based on both maximum-likelihood and Tajima's 1D relative rate tests. When rate constancy was rejected, annuals exhibited higher rates of nucleotide substitution in most cases. Categorical treatment of branch length differences indicated that an excess number of annual species had higher rates of nucleotide substitution. Because these two patterns are expected under the generation time hypothesis for plants, these results support the hypothesis that differences in the annual/perennial habit are associated with rates of molecular evolution in angiosperms.

The simulations reported in this paper supply several insights. First, the simulations showed how often relative rate tests based on *ITS*-like sequences reject the null hypothesis of rate constancy when rates are in fact unequal. Second, the simulations showed how frequently a categorical comparison of branch lengths detects faster substitution rates even when relative rate tests do not reject the null hypothesis. Third, the simulations provide context for observations of *ITS *substitution rate homogeneity or heterogeneity reported in earlier studies, in particular, why substitution rates may not have been associated with differences in annual/perennial habit [24,35]. Because *ITS *sequences are short and have few diverged sites when compared between recently diverged taxa, relatively low power to reject rate constancy seemed possible. Indeed, the simulations showed that the statistical power to detect rate heterogeneity using *ITS *sequences is generally low for rate differences less than 3-fold. For simulations based on *ITS*1-like and *ITS*2-like nucleotide substitution parameter sets, the relative rate test only achieved an 80% probability of rejecting rate homogeneity with 4.5 or 5-fold rate differences. These power analyses for *ITS*-like sequences agree with a more general previous study that demonstrated a high type II error for Tajima's 1D test when the DNA sequences compared are short and have few diverged sites [38]. The simulations further suggest that categorical treatment of branch length differences is a more powerful indicator of rate heterogeneity at low to moderate substitution rate differences compared to relative rate tests, at least for *ITS*-like sequences. However, conclusions about the statistical power of the categorical rate comparisons only apply to the average nucleotide substitution model and divergence parameters used in the simulations and may not be a general phenomenon.

The best-documented case of a generation time effect is the 2- to 3-fold faster substitution rate in rodents compared to hominids [12,7]. This well studied example provides some perspective on the magnitude of rate differences we might expect to observe in plants if a generation time effect actually operates. In plants, the magnitude of rate differences between annual and perennial taxa was 2-fold at synonymous sites in the mitochondrial *coxI *gene [40], 4-fold at both synonymous and non-synonymous sites in the chloroplast *rbc*L gene [20,21,27], and 2.5-fold at synonymous sites in nuclear *Adh *loci [20] (Eyre-Walker and Gaut 1997). All of these studies were limited to comparisons of highly divergent annual and perennial species and therefore confounding factors other than differences in generation time might have lead to an overestimation of the impact of annual/perennial habit on substitution rates. In a study focused on phylogenetically independent comparisons, Kay and collaborators [35] found that clades with a predominantly herbaceous life history exhibited divergence rates for *ITS *sequences almost two times faster than clades with a predominantly long-lived woody life history in 28 phylogenies representing 21 different angiosperm families. The overall substitution rate differences estimated for the annual/perennial species comparisons in this paper were of similar magnitude to the *ITS *rate differences observed by Kay et al. [35]. Here, annuals evolved on average 1.6 times faster rate than perennials when a less divergent outgroup was used, while a slightly lower 1.4-fold average acceleration of annuals was observed with more divergent outgroup taxa. Categorical comparisons for *ITS *sequences also indicated that faster rates of substitution were correlated with annual habit. Therefore, the *ITS *results in this paper support a weak substitution rate acceleration for annuals consistent with a generation time effect in plants. We also believe that our sampling methods controlled for rate heterogeneity caused by variables other than annual/perennial habit and helped to better detect a weak generation time effect. This explains in part why our results are distinct from those of Whittle and Johnston [24], even though both studies were based on some of the same *ITS *data and used similar relative rate tests.

The *Arabidopsis *sequence data also support a weak annual acceleration in substitution rates. The annual *A. thaliana *had a significantly faster substitution rate for the chloroplast *rbc*L locus when compared with *A. petraea*. In addition, categorical rate comparisons consistently showed a faster substitution rates for *A. thaliana *for all the nuclear loci and chloroplast regions, even though rate constancy was not rejected by relative rate tests. When the three nuclear loci were combined, *A. thaliana *had a significantly faster substitution rate than either perennial. The only exception to the pattern of faster divergence rates for *A. thaliana *were at *ITS *sequences. The lack of any relative rate tests rejecting rate homogeneity and about half of qualitative comparisons indicating annuals were faster, all suggest that the *ITS *sequences showed no evidence of a faster substitution rate for *A. thaliana*. However, the simulation results showed that *ITS*-like sequences have little power to reject rate constancy when substitution rates are less than 2-fold different. So the pattern of about half of the qualitative rate comparisons showing a faster substitution rate for annuals is consistent with random variation about a mean rate difference of zero. Interestingly, similar results indicating a consistently higher number of synonymous substitutions (but rate constancy was not rejected by Tajima's relative rate test) in *A. thaliana *than in *A. lyrata *were observed in five out of six loci using the closely related outgroup species *Capsella rubella *and *Arabidopsis graba *[41].

Recent divergence of annual/perennial taxa is an advantage when attempting to infer the possible causes of rate heterogeneity because it reduces the number of evolutionary changes that distinguish the taxa in addition to annual/perennial habit. Unfortunately, that advantage may come at the cost of statistical power to detect potential rate heterogeneity. Recent divergence also means that few substitutions have occurred in the two taxa being compared so that the number of nucleotide changes will be small. The *Arabidopsis *data further suggest that statistical power to compare annual/perennial substitution rates is limiting. The individual *Arabidopsis *nuclear loci did not show significant rate heterogeneity. However, the larger sample of changes in the three loci combined showed the approximately 1.4-fold rate difference between annual and perennial was significantly faster for the annual. Since we did not distinguish among synonymous and nonsynonymous sites in the *Arabidopsis *sequences, the significant rate difference is an average across all types of nucleotide sites and reflects the net substitution rate of neutral sites and any sites influenced by positive or negative selection.

In addition to the low power of *ITS *sequences to test relative rate hypotheses, *ITS *sequences may have other limitations that further hamper their ability to detect rate heterogeneity. There is the possibility that the *ITS*1 and *ITS*2 sequences might be subject to different selective pressures [42] resulting in region-specific rates of *ITS *substitution when natural selection is stronger than genetic drift. The *ITS *sequence data in this paper indicated a pattern of annual taxa exhibiting faster substitution rates that was consistent between both *ITS*1 and *ITS*2 regions. Such a pattern is not expected if *ITS*1 and *ITS*2 regions experience locus-specific selection pressures. In addition, it has been hypothesized that incomplete concerted evolution could independently affect rates of molecular evolution at either *ITS*1 or *ITS*2 [30]. Reports of multiple nrDNA haplotypes within individuals are becoming increasingly common [e.g. [43-51]] suggesting that complete concerted evolution should not always be assumed for *ITS *sequences. The different intraspecific nrDNA ribotypes used in the *Arabidopsis *annual/perennial comparisons did in fact change the perception of substitution rates between *ITS*1 and *ITS*2 regions. Either *ITS*1 or *ITS*2 was observed to have the faster substitution rate for annuals depending on the nrDNA ribotypes used in the *Arabidopsis *annual/perennial comparison. Thus, the *Arabidopsis ITS *data suggest that estimates of substitution rates may depend on the nrDNA ribotype employed in comparisons. If selection pressures or polymorphism dynamics have a greater impact on estimates of substitution rates than does a weak generation time effect, any acceleration in the substitution rate of annuals will be difficult to detect with *ITS*.

The underlying biological mechanisms that might cause an acceleration of substitution rates in annuals are still unclear [18,35], although life history features that influence the number of rounds of DNA replication per unit of calendar time are capable of altering the relative substitution rate when mutation rates are constant. Identifying the underlying cause or causes of rate heterogeneity is difficult because variables such as the combined effects of organism size and temperature on metabolic rate [52,53], the influx of environmental energy that may lead to mutation [54], and mating system [55] are potentially confounded with differences in generation time. Annual or perennial habit may itself have a variable relationship to the generation time pertinent to substitution rates. For example, many perennials are able to flower in their first year like annuals while other perennial species may require many years until first flower. The total range of possible generation times in plants is very large since some woody perennials may live for thousands of years. The species in this study all fall at the short generation time end of this range since they are either annuals or short-lived perennials. Therefore, the suggestion of a weak annual/perennial substitution rate difference in our study may not apply if plant groups containing perennials with longer lives and greater time to first flower were compared.

## Conclusion

*ITS *substitution rates in 16 phylogenetically-independent comparisons of annual and perennial taxa and from the combination of nuclear loci and chloroplast genome regions in annual and perennial *Arabidopsis *suggest a modest rate acceleration of less than 2-fold in annuals. These results support an association between rates of nucleotide substitution and annual/perennial habit in plants as expected under the generation time hypothesis. Separately, simulations showed that relative rate tests employing *ITS*-like sequences are not expected to be powerful enough to reject rate homogeneity when substitution rate differences are small. Given that the power of *ITS *sequences to test for generation time effects is very low, the conclusion by Whittle and Johnston [24] that no annual/perennial effect on substitution rates exits seems unwarranted. The small substitution rate differences observed here and in other studies points out that testing the generation time hypothesis among closely related plant species will require multiple loci to achieve sufficient power, as was the case in the now classic examples of animal generation time effects. While their availability in many plant taxa facilitates phylogenetically-independent comparisons, *ITS *sequences by themselves are not likely to be a powerful tool to test hypotheses involving substitution rate heterogeneity. Further studies with greater statistical power have to be carried out before drawing a definitive conclusion about patterns of relative substitution rate heterogeneity in annual and perennial plants and its possible causes.

## Methods

### DNA sequences

Partial nrDNA sequences from 64 different species representing 13 angiosperm plant families were retrieved from GenBank (Table 4). Because rate heterogeneity at nrDNA sequences may have multiple causes, we established three criteria designed to control for artifacts possibly contributing to rate heterogeneity that may obscure any rate variation caused by differences in annual/perennial life history.

**Table 4:** Genbank accession numbers for annual/perennial species pair and outgroup sequences, where the first taxon is the annual and the second taxon is the perennial for each annual/perennial pair, and for outgroups the first taxon listed is less diverged and the second is more diverged.

Family	Annual/perennial pair	Genbank accession	Outgroup	Genbank accession
Brasicaceae	*Arabidopsis thaliana*	AJ232900	*Capsella rubella*	AJ232912
	
	*Arabidopsis lyrata*	AJ232889	*Arabis turrita*	AJ232906

Fabaceae	*Astragalus tener*	AF121697	*Oxytropis pilosa*	AF121759
	
	*Astragalus membranaceus*	AF121675	*Carmichaelia stevensonii*	AF121751
	
	*Lupinus micranthus*	AF007480	*Chamaecytisus mollis*	AF007472
	
	*Lupinus polyphyllus*	AF007496	*Crotalaria podocarpa*	AF007469

Asteraceae	*Bellis annua*	AF490579	*Bellium bellidiodes*	AF490466
	
	*Bellis perennis*	AF493996	*Crinitaria linosyris*	AF046949
	
	*Erigeron annus*	AF118489	*Bellis annua*	AF490579
	
	*Erigeron divergens*	AF118485	*Bidens alba*	U67107
	
	*Machaeranthera canescens*	U97622	*Aster kingii*	AF515597
	
	*Machaeranthera tanacetifolia*	AF251567	*Erigeron annus*	AF118489

Portulacaceae	*Claytonia parviflora*	AY764042	*Montia parvifolia*	L78034
	
	*Claytonia megarhiza*	L78027	*Calandrinia affinis*	L78020

Polemoniaceae	*Collomia heterphylla*	AF020703	*Gilia stellata*	AF208212
	
	*Collomia rawsoniana*	AF208201	*Allophyllum integrifolium*	AF208199

Geraniaceae	*Erodium alnifolium*	EF185391	*California macrophyllum*	EF185338
	
	*Erodium trifolium*	EF185389	*Geranium dissectum*	AY944413

Polemoniaceae^a^	*Linanthus acicularis*	AF119424 AF119450	*Gilia stellata*	AF208212
	
	*Linanthus floribundus*	AF119429 AF119455	*Polemonium viscosum*	AF016051

Solanaceae	*Nicotiana tabacum*	AJ492447	*Anthocercis gracilis*	AJ492457
	
	*Nicotiana obtusifolia*	AJ492430	*Grabowskia duplicata*	AF238982

Rosaceae	*Potentilla norvegica*	U90790	*Polylepis tarapacana*	AJ512778

	*Potentilla dickinsii*	U90785	*Rosa persica*	AJ416468

Ranunculaceae	*Ranunculus sceleratus*	AF323322	*Myosorus minimus*	AJ347913
	
	*Ranunculus circinatus*	AF323321	*Podophyllum hexandrum*	AF328965

Apiaceae	*Sanicula bipinnata*	AF031982	*Sanicula europaea*	AF031964
	
	*Sanicula crassicaulis*	AJ012694	*Eryngium cervantesii*	AF031960

Malvaceae	*Sidalcea calycosa*	AJ304878	*Eremalche parí*	AJ304938
	
	*Sidalcea ranunculacea*	AJ304926	*Napaea dioica*	AJ304940

Poaceae	*Vulpia alopecuros*	AF478491	*Deschampsia cespitosa*	AF532929
	
	*Festuca borderii*	AF303403	*Brachypodium distachyon*	AF303399

^a ^The nrDNA sequences were obtained from the same PCR product but sequenced independently so there are two GenBank entries [66].

The first criterion was that only *ITS*1 and *ITS*2 sequences obtained from the same PCR amplicon were sampled to distinguish between functional and non-functional copies. A functional copy is expected to be under strong selective constraints limiting its substitution rate while a non-functional copy (pseudogene) is expected to exhibit a higher rate of nucleotide substitution when compared to a functionally constrained copy. Nuclear ribosomal DNA regions are usually located in chromosomal regions within nucleolus organizer regions (NORs) in the form of tandemly repeated arrays. Each nrDNA copy is organized into less constrained *ITS*1 and *ITS*2 regions separated by the 163–164 base pair 5.8S region, which is highly conserved when functional copies are compared within genera or between recently diverged genera [42,56]. A rigorous method to detect pseudogenes is to compare estimated divergence at conserved sequence regions with estimated divergence at unconstrained sequence regions [57]. Thus, we compared nucleotide divergence for 5.8S, *ITS*1 and *ITS*2 regions for each set of nrDNA sequences from the annual/perennial/outgroup comparison. We excluded from further analyses nrDNA sequences that exhibited either 1) a high divergence at the 5.8S region relative to the others 5.8S regions, or 2) divergence of the 5.8S region that was approximately equal to divergence at the *ITS*1 and *ITS*2 regions within a species, because these are patterns consistent with a lack of 5.8S functional constraint. This is a conservative sampling approach to prevent inadvertently combining functional and non-functional nrDNA copies in comparisons of annual and perennial taxa that could hamper our ability to detect possible associations between divergence rates and life history. The criterion of using nrDNA sequences from the same PCR amplicon was restrictive in that it caused us to exclude numerous possible ITS sequences available in GenBank.

The second criterion was that pairs of annual/perennial taxa sampled had nrDNA sequences from two outgroup taxa that were relatively closely related within the same family. This permitted examination of the impact of outgroup divergence on relative rate comparisons between annual and perennial taxa and prevented our relative rate estimates and hypothesis tests from being contingent on the peculiarities of a single outgroup.

The third criterion was that each of the *ITS*1 and *ITS*2 sequences were required to have at least eight nucleotide changes between annual and perennial species. Complete *ITS *sequences (*ITS*1, 5.8S and *ITS*2) were approximately 600 base pairs long on average so that eight nucleotide changes in 600 base pairs is equal to 1.3% divergence. Since the power of relative rate tests depends on the number of substitutions, this criterion prevented sampling of sequences likely to have little statistical power to reject the null hypothesis of rate homogeneity.

The annual *Arabidopsis thaliana *and the perennials *A. lyrata *subsp. *lyrata *(*A. lyrata*) and *A. lyrata *subsp. *petraea *(*A. petraea*) met the three sampling criteria for nrDNA and also offered three additional nuclear loci (*Chs*, *Adh *and *Pgi*C) and the *rbc*L and *matK *chloroplast regions (see accession numbers in Table 5). The *Arabidopsis *taxa also offer intra-specific variation in nrDNA ribotypes and consequently polymorphic *ITS*1 and *ITS*2 sequences that permitted us to test whether relative rate comparisons were influenced by nrDNA polymorphism.

**Table 5:** GenBank accession numbers for sequences sampled of the annual *Arabidopsis thaliana*, the two perennials *Arabidopsis lyrata *subspecies *lyrata *and *Arabidopsis lyrata *subspecies *petraea *as well as the outgroup *Crucihimalaya himalaica*.

Taxa	*ITS*	*rbc*L	*matK*	*Chs*	*Adh*	*Pgi*C
*C. himalaica*	AJ232933	D88902	AF144356	AF144531	AB015503	AB080909

*A. thaliana*	AJ232900	D88901	AF144348	AF112086	AF110456	AB044955

*A. lyrata*	AJ232889	AY174645	AF144342	AF112100	AF110449	AY174553

*A. petraea*	AJ232891 (R1)	AY174650	AF144331	AJ619894	AF110452	AY174537

*A. petraea*^a^	AJ232896 (R2)					

^a ^Two *ITS *ribotypes are available for *A. petraea*, here referred to as R1 and R2.

### Alignment and phylogenetic analyses

Sequences were aligned into contigs for each comparison of an annual, perennial and an outgroup taxon and edited using Sequencher 4.5 (Gene Codes, Ann Arbor, Mich.). Gapped positions were pruned from alignments before analyses. Modeltest 3.5 [58] was used to determine the most likely nucleotide substitution model and the associated parameters for each triplet annual/perennial/outgroup sequence comparison. Branch lengths for each annual/perennial comparison were determined for *ITS*1, *ITS*2 and for the combination of both *ITS*1 and *ITS*2 regions (the combined *ITS *region) using PAUP* v.4.0b10 [59] and HyPhy [60] under the parameters of the substitution model determined in Modeltest. To express differences in nucleotide substitution rates between annual/perennial pairs, the substitution rate of the taxon exhibiting longer branch length was divided by the substitution rate of the taxon with the shorter branch length. In addition, the generation time hypothesis has been tested via categorizing branch length differences (e.g. annual faster than perennial or perennial faster than annual) even when rate homogeneity is not rejected by a relative rate test [24]. Thus, annual/perennial branch length differences were also summarized by a categorical variable to indicate whether the annual or perennial species exhibited the higher rate of substitution. Following Whittle and Johnston [24], we performed sign tests of the null hypothesis that annuals and perennials exhibited a longer branch length with equal frequency. In contrast to Whittle and Johnston [24], we employed one-tailed versions of the test because under the alternative hypothesis of rate heterogeneity annuals are expected to have a faster substitution rate than perennials.

### Relative rate tests

To test for differences in substitution rates among taxa, both maximum-likelihood [61] and Tajima's 1D [36] relative rates tests were applied to the *ITS *sequences for each independent annual/perennial species pair. The relative rate test compares the number of nucleotide substitutions that occurred in one of the ingroup species to the number of substitutions that occurred in the other ingroup species utilizing the outgroup to identify those substitutions that can be unambiguously assigned to one of the ingroup taxa [62,63]. Under the null hypothesis of equal substitution rates in each lineage, the number of nucleotide changes is expected to be equal for the two taxa. The maximum-likelihood relative rate test is considered one of the most powerful and flexible tests for rate heterogeneity, but it requires knowledge of the nucleotide substitution pattern, any substitution rate variation among sites in addition to the phylogenetic relationship among sequences [27]. The maximum-likelihood relative rate tests were implemented in HyPhy [60] and used the nucleotide substitution models from Modeltest.

An alternative relative rate test which does not require an explicit nucleotide substitution model is Tajima's 1D test [36]. Although Tajima's 1D test cannot correct for saturation, apparent divergences are not expected to be gross under-estimates of true divergences for recently diverged taxa. The null hypothesis of rate constancy can be tested with Tajima's 1D using a chi-square with one degree of freedom as implemented in the T1Dand2D v4OS program [64]. Because sites with gaps or ambiguous base calls can be considered as an additional change by the Tajima's 1D program, they were excluded from the analyses.

### Computer simulations

We investigated the power of maximum likelihood relative rate tests for each of the *ITS*1, *ITS*2 and combined *ITS *regions by computer simulation by utilizing empirically estimated sequence substitution parameters. Simulation parameters were based on nucleotoide substitution parameters estimated from *ITS *sequences and were divided into two groups based on divergence of the outgroup within each annual/perennial pair (Table 2). Transition/transversion ratio, sequence length and substitution rate difference between annuals and perennials were averaged over all independent annual/perennial pairs. Because the nucleotide substitution models for annual/perennial/outgroup triplets were somewhat variable (see Results), the most frequently obtained nucleotide substitution model was employed in the simulations.

The combination of SG Runner (T. Wilcox; homepage.mac.com/tpwilcox/) and Seq-Gen [65] were used to model each of the *ITS *regions. Seq-Gen simulates nucleotide substitution within lineages until a given threshold of divergence between the ingroup taxa has been reached. This threshold divergence value was obtained by averaging the estimated divergences (or branch lengths) from all annual/perennial/outgroup triplets (see Figure 1). Each *ITS*-like data consisted of 1000 DNA sequence triplets simulated with one of the ingroup taxon having a substitution rate between 1.5 and 5 times faster than the other ingroup taxon. The threshold branch length values of the taxon with the slower substitution rate parameter sets (also denoted as the perennial-like taxon) and the outgroup taxa were kept constant. The threshold branch length value of the taxon with the faster substitution rate parameter sets (also denoted as the annual-like taxon) was simulated with a rate difference of 1.5 to 5 times increasing in steps of 0.5 times the threshold branch length value of the perennial-like taxon.

Each set of triplet sequences was analyzed in PAUP* to calculate the relative branch lengths and the maximum likelihood values of each constrained and unconstrained tree. Then, a likelihood ratio test (LRT) was carried out for each of the 1000 replicates using an Excel spreadsheet and used to calculate the proportion of replicates where the null hypothesis of rate constancy was rejected. The percent of cases where the LRT rejected rate constancy was divided into instances where either the faster evolving annual-like taxon or the slower evolving perennial-like taxon had the longer estimated branch length. In addition, branch length differences in each replicate simulation were categorized into qualitative outcomes of annual-like taxon faster or perennial-like taxon faster, independent of whether or not the relative rate test rejected rate constancy. This provided an estimate of the proportion of replicates where the categorical comparison of rates detected rate heterogeneity. In order to evaluate the variation in estimates of annual/perennial rate differences in sequences most similar to actual *ITS *data, one set of 1000 replicate simulations were carried out using the nucleotide substitution model parameters and average rate differences estimated from the *ITS *sequences of the 16 annual/perennial pairs (see bottom rows of Table 2). The distribution of the estimated rate differences between the ingroup taxa in 1000 replicate triplet sequences was plotted in histograms for the six combinations of three *ITS *nucleotide substitution models and more and less diverged outgroups.

## Authors' contributions

The four authors jointly conceived and designed the study. DSH and OFP assembled the data and performed the analyses. JMB and MBH coordinated the study. MBH and DSH wrote the manuscript. All authors read, edited and approved the final manuscript.
